# Premature closure of the distal radial physis without evident history of trauma: A case report

**DOI:** 10.1097/MD.0000000000021515

**Published:** 2020-07-31

**Authors:** Yuji Tomori, Mitsuhiko Nanno, Shinro Takai

**Affiliations:** a Department of Orthopaedic Surgery, Nippon Medical School Musashi Kosugi Hospital, Kanagawa; b Department of Orthopaedic Surgery, Ukima Central Hospital; c Department of Orthopaedic Surgery, Nippon Medical School Hospital, Tokyo, Japan.

**Keywords:** premature closure, growth arrest, distal radial physis, distal radius, case report

## Abstract

**Rationale:**

The distal radius is the region of the body with the highest incidence of physeal injury. However, it is uncommon for the distal radius to undergo growth arrest without a history of trauma. We present a case of premature closure of the distal radial physis without evident history of trauma in a girl.

**Patient concerns:**

A 14-year-old girl presented with chronic progressive deformity and painful functional limitation of her right forearm. The right wrist pain had begun when the patient was 5 years old. There was no evident history of trauma. The deformity and persistent right wrist pain had prevented her from performing sports activities and activities of daily living.

**Diagnoses:**

Radiography and computed tomography showed a volarly angulated distal radius and dorsally protruding distal ulna with a length discrepancy between the distal radius and ulna due to premature physeal closure of the right distal radius.

**Interventions:**

To eliminate the deformity and achieve painless functional recovery of the wrist, an opening wedge osteotomy of the distal radius with an iliac bone graft was performed, followed by a shortening osteotomy of the distal ulna.

**Outcomes:**

Radiography at final follow-up 1 year and 9 months postoperatively showed good alignment of the distal radioulnar joint without length discrepancy between the two forearm bones. The range of motion of the left wrist had reached 100% of the contralateral wrist without any pain, and the right grasp strength was 18 kg, which was 82% compared with the dominant left wrist.

**Lessons:**

Premature closure of the distal radial physis impairs the growth potential of the physis and leads to wrist dysfunction due to deformities. In the present case, a satisfactory outcome was achieved via corrective osteotomy of the distal radius with an iliac bone graft combined with ulnar shortening osteotomy.

## 1 Introduction

Premature closure of the distal radial physis is a rare condition that results in the impairment of the growth potential of the physis.^[1–3]^ These cases have a poor prognosis and common sequelae include radioulnar discrepancy and dorsal subluxation or dislocation of the ulna, limited range of motion of the wrist, and loss of grasp strength.^[1,2,4,5]^ The reported causes of premature physeal closure are trauma, decreased blood supply, immobilization, sepsis, tuberculosis, poliomyelitis, and vitamin A toxicity.^[1,2,5]^

We present a case of a 14-year-old girl with idiopathic premature closure of the distal radial physis. To the best of our knowledge, this is the first case report of a patient with premature closure of the distal radial physis without a known cause.

## 2 Case report

A 14-year-old girl presented to our hospital because of deformity and painful functional limitation of her right forearm that had been developing over many years. The right wrist pain had started when she was 5 years old. She had since experienced progressive deformity and functional loss that prevented her from performing sports activities and activities of daily life. The patient was a left-handed junior high school student who had played tennis 6 days per week for 2 years, and had occasionally played basketball for about 8 years. She had no history of trauma to the right wrist, including physeal fracture or bone bruising. She had a systemic history of vascular purpura at 6 years old and absence epilepsy at 12 years old; she had been taking antiepileptic medication (sodium valproate), but not corticosteroids.

**Figure 1:**
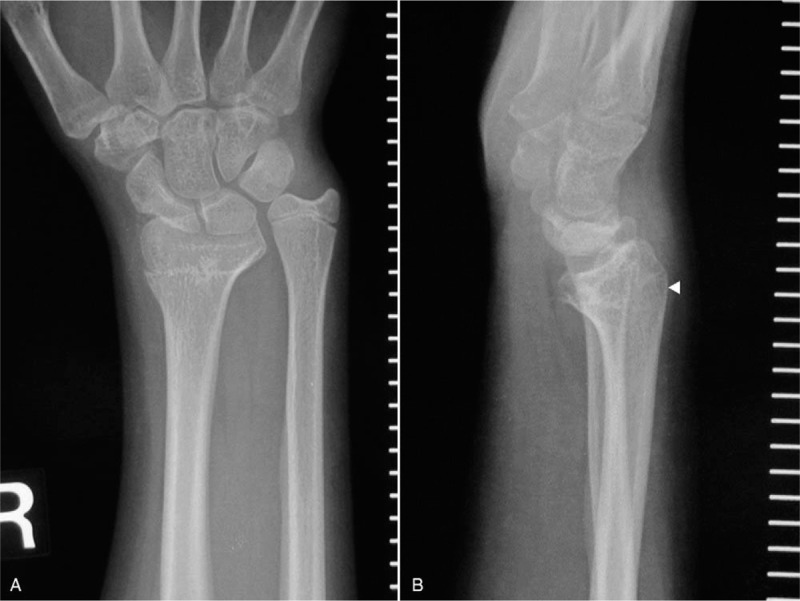
Radiographs taken at the time of presentation showing the right forearm of a 14-year-old girl with painful functional limitation due to radioulnar discrepancy and dorsal subluxation of the ulna. (A) Anteroposterior view. (B) Lateral view.

**Figure 2:**
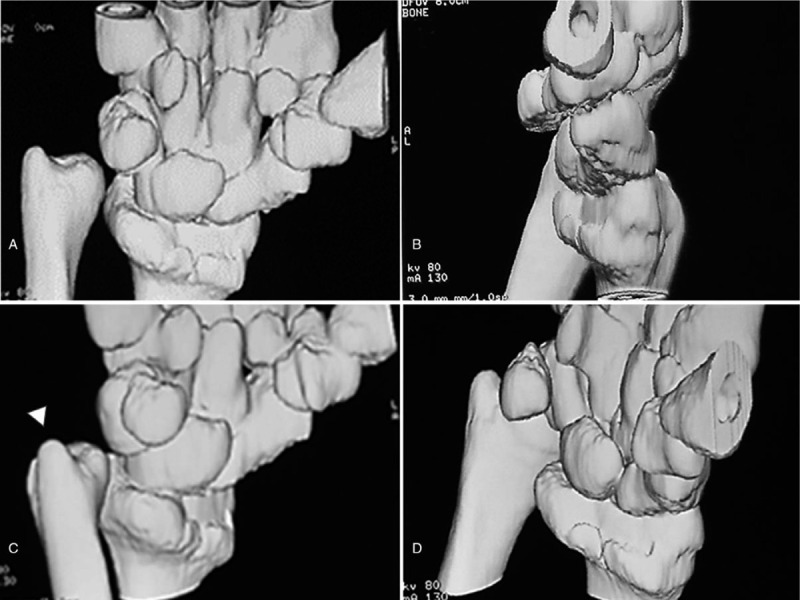
Three-dimensional computed tomography images of the distal forearm showing subluxation of the ulnar head to the posterior side of the sigmoid notch on the radius (arrowhead) and a volarly angulated distal radius.

Physical examination showed mild swelling of the dorsum of the right wrist and dorsal protrusion of the distal ulna compared with the contralateral wrist, with the appearance resembling a Madelung-like deformity. The right wrist was able to achieve extension of 90° and flexion of 65°, and the right forearm had a full range of rotation. The right wrist had 97% range of motion compared with the contralateral wrist. Plain radiographs showed angulation of the distal radius and early closure of the physis of the distal radius, resulting in the ulnar length discrepancy (Fig. 1A and B). The ulnar inclination, volar tilt of the distal radius, and ulnar variance of the affected wrist vs the contralateral wrist were 10° vs 28°, 35° vs 12°, and +12 mm vs 0 mm, respectively. Three-dimensional computed tomography revealed volar angulation of the distal radius, dorsal protrusion of the distal ulna and radius, and a radioulnar length discrepancy (Fig. 2A–D). Six months after the initial visit to our hospital, the patient provided written informed consent for surgery and for the publication of her anonymized images in this case report.

**Figure 3:**
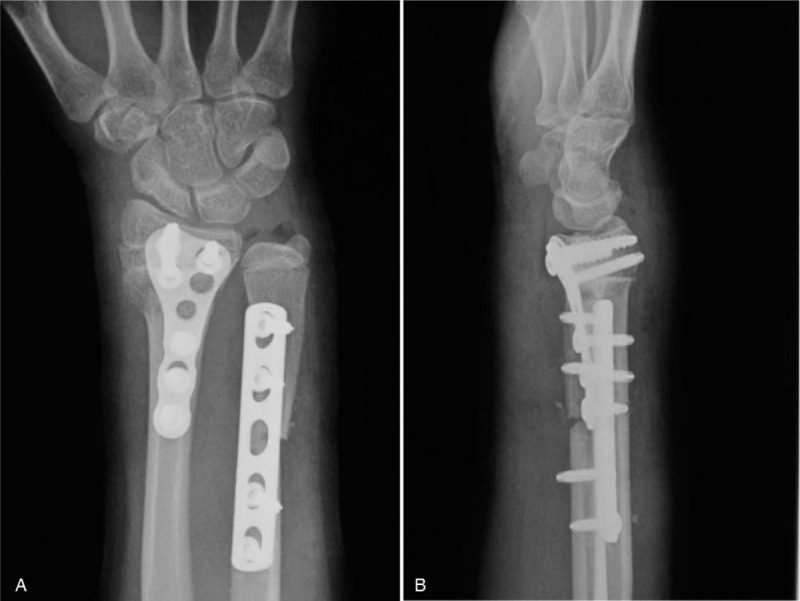
Postoperative radiographs of the right wrist. (A) Anteroposterior view. (B) Lateral view. The dorsal angulation of the distal radius was corrected and fixed with a volar locking plate. Ulnar shortening osteotomy was performed, followed by fixation of the locking plate.

To eliminate the deformity and to achieve painless functional recovery of the wrist, the patient underwent opening wedge osteotomy of the distal radius with an iliac bone graft followed by distal ulnar shortening osteotomy. Briefly, the patient was placed in the supine position with the affected limb positioned to expose the surgical site, and an air tourniquet was applied. The distal radius deformity was treated via opening wedge osteotomy with an iliac bone graft and internal fixation with a volar locking plate. The distal radius was explored through a radial longitudinal incision above the flexor carpi radialis, with the flexor tendon retracted to the ulnar side. The flexor pollicis longus was exposed and retracted radially to expose the pronator quadratus. The pronator quadratus was divided and elevated to reveal the deformation site. At 1 cm distal to the distal end of the radius, opening wedge osteotomy was carried out to adjust the volar tilt and length of the radius. A block of iliac bone was harvested and shaped to fit the radial defect, followed by fixation with a volar locking plate. Subsequently, distal ulnar shortening osteotomy was carried out at 5 cm distal to the distal end of the ulna, followed by fixation with a locking plate. Postoperative radiographs showed that the radiocarpal and distal radioulnar joints were well aligned. The angle of radial inclination, volar tilt, and ulnar variance were 22°, 16°, and −6 mm, respectively (Fig. 3A and B).

**Figure 4:**
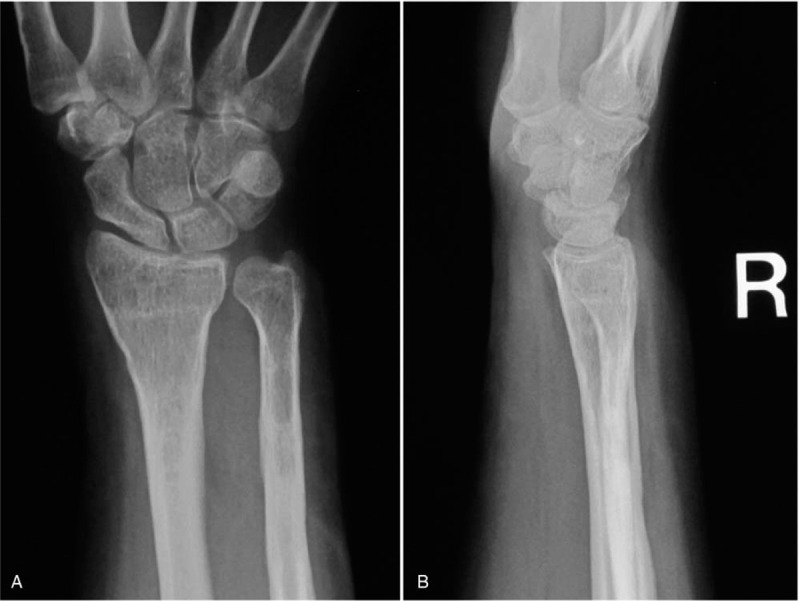
Presenting anteroposterior (A) and lateral (B) radiographs of her right wrist at a year and 9 months after the operation. The radiocarpal and distal radioulnar joint was restored.

Postoperatively, a short-arm splint was applied for 4 weeks. Active range of motion exercises of the right wrist were permitted beginning the day after the removal of the splint. Surgical treatment resulted in pain relief, improvement of the mobility arc, full rotation of the forearm, and the resumption of the patient's regular activities within 3 months. A 6 months postoperatively, plain radiography showed complete union of both bones. Thus, a second surgery was performed to remove the plates and screws. Radiographs obtained at final follow-up showed good alignment of the distal radioulnar joint without any length discrepancy between the two forearm bones (Fig. 4A and B). The right ulna had gained about 1 cm of growth, but the growth plate of the distal ulna was closed at 1 year and 9 months postoperatively. The angle of radial inclination, volar tilt, and ulnar variance were 21°, 14°, and 0 mm. The right wrist showed extension of 90°, flexion of 70°, and a full range of forearm rotation. The range of motion of the right wrist was 100% compared with the contralateral wrist without pain. The grasp strength of the right hand was 18 kg, which was 82% compared with the dominant left hand. Although the right radius was 1 cm shorter than the unaffected side, the deformity of the wrist had been corrected, and the patient was satisfied with the outcome.

## 3 Discussion

The epiphysis of the distal radius begins to ossify at 8 to 18 months of age and closes at ∼17 years, with closure occurring earlier in girls than in boys.^[2]^ The growth plate in the growing skeleton is vulnerable and occasionally incurs direct epiphyseal damage due to physeal fracture, axial loading trauma, repetitive axial trauma, epiphyseal ischemia, infection, immobilization, or surgical interference.^[1,2,4–6]^

In the present case, although radiographs showed physeal thinning and subjacent mild metaphyseal sclerosis in the central and radial aspects of the distal radial physis that indicated an associated Salter Harris type V injury,^[7,8]^ there was no history of trauma to the wrist, including epiphyseal fracture or bone bruising. Furthermore, the pathology was unilateral, and the physical examination did not suggest ischemia due to immobilization, vascular injuries, or infection of the wrist. Thus, the cause of the growth arrest in the present case was unknown. To the best of our knowledge, this is the first reported case of a patient with premature closure of the distal radial physis without any evident cause.

The mechanism that led to premature closure of the distal radial physis in the present case is unknown; however, the possibilities include repetitive axial pressure damage or occult physeal injury to the distal radius epiphysis. Repetitive axial stress to the physis of the distal radius can lead to growth disturbance and is frequently seen in young gymnasts who bear the full weight of their bodies.^[9]^ Weightbearing gymnastic activities transmit the body weight through the wrist, and repetitive compression stress across the physis causes permanent growth arrest of the distal radius due to early closure of the physis.^[2,9]^

Although the present patient had no history of wrist trauma, there are several reports of growth arrest of the distal radius following physeal or metaphyseal fracture.^[1,4]^ Posttraumatic early closure of the physis of the distal radius due to Salter-Harris type I, II, and V fractures is extremely rare, but previous physeal injury of the distal radius might cause growth plate arrest.^[1,2,4,5]^

The present patient was a tennis and basketball player. Most injuries result from acute trauma, and wrist injuries typically occur during contact with the ball while serving or receiving in tennis, and while throwing a ball in the overhand pass position or while blocking in basketball. In the present case, the physeal injury of the distal radius might have occurred as a result of repeated trauma caused by contact with the ball during overhand passing, resulting in a Salter-Harris type V injury.

As early growth arrest of the distal radius results in severe deformity, surgical intervention for this deformity is considered necessary to eliminate pain and restore mobility, as well as for cosmetic reasons.^[2,10]^ For patients whose growth has already finished, the deformity is corrected via lateral opening or closing wedge osteotomy of the radius and/or an ulnar shortening osteotomy to maintain the distal radio-ulnar function.^[2]^ However, when the growth arrest occurs before growth is completed, the condition is more challenging to treat and post-treatment recurrences are common. Surgical options for premature closure of the physeal plate include corrective osteotomy, epiphysiodesis, and bone bridge resection.^[2,10]^ There are several reports of angular deformity being successfully corrected via osteotomy and ulnar shortening.^[6]^ This method is applicable when the bone is approaching maturity and there is little remaining growth. In the present case, the bone had almost matured and only little growth remained. Thus, we performed an opening osteotomy with an iliac bone graft on the radius. This technique allows the deformity to be corrected by placing the forearm in the required functional arc without causing shortening of the osteotomized segment. However, an ulnar shortening osteotomy was also required because the opening corrective osteotomy of the distal radius was not sufficient to restore the distal radioulnar joint. Although growth arrest of the distal radius in children rarely occurs, opening osteotomy with an iliac bone graft for the distal radius combined with ulnar shortening osteotomy provided excellent grip strength and maintained the normal morphology of the wrist.

## 4 Conclusion

Premature closure of the distal radial physis is a rare condition that impairs the growth potential of the physis and leads to wrist dysfunction due to deformities. Although several causes have been reported for premature closure of a physis, to the best of our knowledge, there are no reported cases of idiopathic premature closure of the distal radial physis. In the present case, a satisfactory outcome was achieved after corrective osteotomy of the distal radius with an iliac bone graft combined with ulnar shortening osteotomy.

## Acknowledgments

We thank Kelly Zammit, BVSc, from Edanz Editing (www.edanzediting.com/ac), for editing a draft of this manuscript.

## Author contributions

**Investigation:** Yuji Tomori.

**Writing – original draft:** Yuji Tomori.

**Writing – review & editing:** Yuji Tomori, Mitsuhiko Nanno, Shinro Takai.
